# Generation of Cellular Reactive Oxygen Species by Activation of the EP2 Receptor Contributes to Prostaglandin E2-Induced Cytotoxicity in Motor Neuron-Like NSC-34 Cells

**DOI:** 10.1155/2020/6101838

**Published:** 2020-01-11

**Authors:** Yasuhiro Kosuge, Hiroshi Nango, Hiroki Kasai, Takuya Yanagi, Takayuki Mawatari, Kenta Nishiyama, Hiroko Miyagishi, Kumiko Ishige, Yoshihisa Ito

**Affiliations:** Laboratory of Pharmacology, School of Pharmacy, Nihon University, 7-7-1 Narashinodai, Funabashi-shi, Chiba 274-8555, Japan

## Abstract

Amyotrophic lateral sclerosis (ALS) is a devastating motor neuron disease characterized by progressive degeneration of motor neurons in the central nervous system. Prostaglandin E2 (PGE2) plays a pivotal role in the degeneration of motor neurons in human and transgenic models of ALS. We have shown previously that PGE2 directly induces neuronal death through activation of the E-prostanoid (EP) 2 receptor in differentiated NSC-34 cells, a motor neuron-like cell line. In the present study, to clarify the mechanisms underlying PGE2-induced neurotoxicity, we focused on generation of intracellular reactive oxygen species (ROS) and examined the effects of N-acetylcysteine (NAC), a cell-permeable antioxidant, on PGE2-induced cell death in differentiated NSC-34 cells. Dichlorofluorescein (DCF) fluorescence analysis of PGE2-treated cells showed that intracellular ROS levels increased markedly with time, and that this effect was antagonized by a selective EP2 antagonist (PF-04418948) but not a selective EP3 antagonist (L-798,106). Although an EP2-selective agonist, butaprost, mimicked the effect of PGE2, an EP1/EP3 agonist, sulprostone, transiently but significantly decreased the level of intracellular ROS in these cells. MTT reduction assay and lactate dehydrogenase release assay revealed that PGE2- and butaprost-induced cell death were each suppressed by pretreatment with NAC in a concentration-dependent manner. Western blot analysis revealed that the active form of caspase-3 was markedly increased in the PGE2- and butaprost-treated cells. These increases in caspase-3 protein expression were suppressed by pretreatment with NAC. Moreover, dibutyryl-cAMP treatment of differentiated NSC-34 cells caused intracellular ROS generation and cell death. Our data reveal the existence of a PGE2-EP2 signaling-dependent intracellular ROS generation pathway, with subsequent activation of the caspase-3 cascade, in differentiated NSC-34 cells, suggesting that PGE2 is likely a key molecule linking inflammation to oxidative stress in motor neuron-like NSC-34 cells.

## 1. Introduction

Amyotrophic lateral sclerosis (ALS) is a type of motor neuron disease characterized by progressive atrophy of skeletal muscle resulting from selective degeneration of motor neurons. The molecular mechanisms underlying this selective vulnerability are still unknown, but inflammation is considered to be an important factor contributing to the pathogenesis of both patients and animal models of ALS [1–3]. Prostaglandins are small lipid inflammatory mediators derived from arachidonic acid via multienzymatic reactions. Five primary prostaglandins are synthesized *in vivo*—prostaglandin D2 (PGD2), prostaglandin E2 (PGE2), prostaglandin F2, prostaglandin I2, and thromboxane A2—and their biological functions occur through seven distinct transmembrane-spanning G-protein-coupled receptors (GPCRs), termed D-prostanoid receptor (DP) 1 and 2, E-prostanoid receptor (EP) 1 to 4, F-prostanoid receptor (FP), I-prostanoid receptor (IP), and thromboxane-prostanoid receptor (TP) [4, 5]. Among them, PGE2 plays a pivotal role in the degeneration of motor neurons in humans and transgenic models of ALS. PGE2 is increased in cerebrospinal fluid from patients with sporadic ALS [6] and in the spinal cord of ALS model mice [7]. We have revealed that the level of microsomal PGE synthase-1 (mPGES-1), the enzyme catalyzing the final step of PGE2 biosynthesis, is significantly increased in motor neurons in the mouse model of ALS [8]. Importantly, inhibition of mPGES-1 by AAD-2004, a dual-function drug derived from aspirin and sulfasalazine, has been reported to exhibit significant neuroprotective effects and to prolong survival in ALS model mice [9]. More recently, we reported that positive-feedback regulation of EP2 in spinal motor neurons may exacerbate PGE2-induced damage to neurons during the progression of ALS in the murine model [10]. These results suggest that PGE2-EP2 signaling is a critical mediator of motor neuron death in the pathogenesis of ALS.

A growing amount of evidence now suggests that oxidative stress contributes to motor neuronal death in ALS [11]. In fact, edaravone, a free radical scavenger, was approved as a therapeutic drug for ALS in 2015 in Japan and South Korea and in 2017 in the United States [12]. More importantly, oxidative stress and chronic inflammation are linked and perpetuate each other, resulting in progression of a number of neurodegenerative diseases including ALS [13, 14]. Oxidative stress leads to exacerbated inflammatory responses, and, conversely, uncontrolled inflammation is known to cause cellular accumulation of reactive oxygen species (ROS), which are associated with oxidative stress [15, 16]. Several previous observations have also indicated that PGD2 and its J2 series metabolites (such as PGJ2, *Δ*12-PGJ2, and 15d-PGJ2) act as potential inducers of intracellular oxidative stress [17–19]. Especially, J2-series prostaglandin-induced ROS production is well correlated with cytotoxicity in a human neuroblastoma cell line, SH-SY5Y [17]. Despite numerous studies suggesting important roles for PGE2 in neuron cell death, very little is known about the ability of PGE2 to act as a ROS inducer/source in motor neurons. Earlier studies at our laboratory revealed that PGE2 directly induces cell death in differentiated NSC-34 cells, which possess the unique morphological and physiological characteristics of motor neurons [20], and that the neurotoxic effect of PGE2 is mediated by activation of EP2 in motor neurons [10, 21]. In the present study, to identify endogenous inducers of intracellular oxidative stress and clarify the molecular mechanism underlying the interaction of oxidative stress with the inflammatory response in ALS neurodegeneration, we examined the ability of PGE2 to induce intracellular ROS production in the mouse motor neuron-like cell line NSC-34, and found that EP2 receptor-dependent ROS production contributes to PGE2-induced cytotoxicity.

## 2. Materials and Methods

### 2.1. Cell Culture and Reagents

NSC-34 cells were seeded at a density of 25,000 cells/cm^2^. To enhance cell differentiation, the medium was exchanged for Dulbecco's modified Eagle's medium/Nutrient Mixture F-12 1:1Mixture (Life Technologies Corporation, Carlsbad, CA, USA) containing 0.5% fetal bovine serum (FBS; Life Technologies Corporation), 1% (*v*/*v*) MEM nonessential amino acid solution (100X) (Life Technologies Corporation), and 1% (*v*/*v*) penicillin-streptomycin solution (100X) (Life Technologies Corporation) in a humidified atmosphere containing 5% CO_2_ at 37°C. PGE2 (Tokyo Chemical Industry Co., Ltd., Tokyo, Japan), butaprost (Cayman Chemical, Ann Arbor, MI, USA), sulprostone (Cayman Chemical), PF-04418948 (Cayman Chemical), and L-798,106 (Cayman Chemical) were dissolved in dimethyl sulfoxide (DMSO; Sigma-Aldrich, St. Louis, USA). N-acetyl-L-cysteine (NAC; Sigma-Aldrich) and dibutyryl-cAMP (Daiichi Sankyo, Tokyo, Japan) were dissolved in water. Untreated NSC-34 cells were used as controls for all our comparative analyses.

### 2.2. MTT Reduction Assay

Cell viability was determined by 3-(4,5-dimethylthiazol-2-yl)-2,5-diphenyl tetrazolium (MTT) reduction assay in 96-well plates as described previously [21]. Briefly, the cells were incubated with MTT (0.25 mg/mL) for 3 h at 37°C. The MTT formazan product was solubilized by adding a solution containing 50% dimethylformamide and 20% sodium dodecyl sulfate (SDS) (pH 4.7), and its amount was determined by measuring the absorbance with a microplate reader SH-1000Lab (Corona Electric, Ibaraki, Japan) at a test wavelength of 570 nm and a reference wavelength 655 nm. The relative cell viability was calculated as the percentage of untreated cells.

### 2.3. LDH Assay

The extent of cytotoxicity was quantified by measurement of lactate dehydrogenase (LDH) released into the medium during exposure to drugs. After NSC-34 cells had been incubated with PGE2 for 48 h, the supernatants were collected in new plates and LDH release was determined using a LDH-Cytotoxic Test kit (Wako Pure Chemical Industries) as described previously [22]. The absorbance of samples was measured at 570 nm using a microplate reader SH-1000Lab (Corona Electric). The background absorbance obtained from the culture medium was subtracted from the absorbance of each sample. The LDH release in each treatment group was calculated as a percentage of the LDH release from the cells treated with 0.2% Tween-20. In all cases, cell death was confirmed by microscopy.

### 2.4. Live/Dead Assay

Amount of viable and dead cells was evaluated with LIVE/DEAD® Viability/Cytotoxicity Kit for mammalian cells (Molecular Probes, Eugene, OR, USA) according to the manufacturer's instructions. Briefly, 4 *μ*M calcein-AM and 2 *μ*M ethidium homodimer-1 (EthD-1) were added to culture medium for 40 min. The images were collected with inversed fluorescence microscope (IX70, Olympus, Tokyo, Japan). Cell mortality was determined by calculating the percentage of EthD-1-positive cells to the total number of cells (the sum of calcein-positive live cells and EthD-1-positive dead cells).

### 2.5. Measurement of Intracellular ROS

Generation of ROS was evaluated by measurement of dichlorofluorescein (DCF) fluorescence as described previously [23]. The cells were preincubated with a standard artificial cerebrospinal fluid (aCSF; 136 mM NaCl, 5 mM KCl, 2.5 mM CaCl_2_, 0.5 mM MaCl_2_, 10 mM HEPES, 10 mM glucose, and 12 mM NaHCO_3_, pH 7.4 buffered by Tris) containing 1 *μ*M 2′,7′-dichlorodihydrofluorescein diacetate (DCFH-DA; Life Technologies Corporation) and 25 *μ*g/mL Hoechst 33258 (Sigma-Aldrich) at 37°C for 60 min. The cells were washed with the medium and then exposed to drugs. The cells were then washed with aCSF, and the DCF fluorescence intensities were measured at excitation and emission wavelengths of 485 and 520 nm, respectively, using a fluorescence plate reader (FLUOstar OPTIMA, BMG Lab Technologies, Germany). Hoechst 33258 fluorescence was measured at excitation and emission wavelengths of 355 and 460 nm. The intracellular ROS level was calculated as the DCF fluorescence intensity normalized to the number of cells, as determined from the Hoechst 33258 fluorescence intensity as described previously [24]. The results are expressed as fold change relative to vehicle-treated cells which were assigned a value of 1.

### 2.6. Western Blotting

NSC-34 cells were plated at a density of 2.0 × 10^5^ cells per 35 mm dish. Western blot analysis was performed as described previously [21, 25]. Briefly, differentiated NSC-34 cells were harvested in a lysate buffer containing 150 mM NaCl, 1% Nonidet P-40, 0.5% sodium deoxycholate, 0.1% SDS, 50 mM Tris-HCl pH 8.0, 1% Triton, 5 mM EDTA, and protease inhibitor cocktail (Roche, Switzerland). Protein extracts were separated on SDS-polyacrylamide gel with Tris/glycine running buffer. After electrophoretic separation, the proteins were transferred to polyvinylidene difluoride (PVDF) membranes (Millipore, USA). The membranes were blocked in blocking buffer (20 mM Tris-HCl pH 7.6, 137 mM NaCl, 0.05% Tween-20, 5% skim milk) for 1 h at room temperature and then treated with a polyclonal antibody against pro-caspase-3 (1 : 1000; Cell Signaling Technology, Danvers, MA, USA) and cleaved caspase-3 (1 : 1000; Cell Signaling Technology), and a monoclonal antibody against *β*-actin antibody (Sigma-Aldrich) overnight at 4°C. The membranes were washed repeatedly in Tris-buffered saline (20 mM Tris-HCl pH 7.6, 137 mM NaCl) containing 0.05% Tween-20, and then a HRP-conjugated secondary antibody (Santa Cruz Biotechnology, USA) was added for 1 h. Immunoreactive bands were visualized with enhanced chemiluminescence (ECL) Western blotting detection reagents (GE Healthcare, Little Chalfont, Buckinghamshire, UK). The blots are scanned, and the optical density of the blots was measured using Scion imaging analysis software (Scion, Frederick, MD, USA). Quantitative results were expressed as the ratio of the band intensity of the protein of interest relative to the band intensity of *β*-actin.

### 2.7. Extraction of Total RNA and Real-Time Semiquantitative PCR Analysis

Gene expression of the EP3 isoforms was determined by semiquantitative real-time RT-PCR as described previously with modifications [26]. Briefly, total RNA was extracted using a High Pure RNA Isolation kit (Roche Diagnostics, GmbH, Penzberg, Germany) in accordance with the manufacturer's instructions. After treatment with DNase I, cDNA was synthesized using Oligo (dT)18 primers and a Transcriptor First Strand cDNA Synthesis Kit (Roche Diagnostics). Aliquots of cDNA were amplified on a MX3000P real-time PCR system (Stratagene, La Jolla, CA, USA) using SYBR® Premix Ex Taq™ II (Takara Biotechnology, Shiga, Japan). All samples were assayed in duplicate. The levels of mRNA expression were normalized to an internal standard (*β*-actin; *ΔΔ*CT method). The mouse primer sequences were 5′-CGG AAG TTC TGC CAG ATC AGA-3′ (forward) and 5′-TCC AGC TGG TCA CTC CAC ATC-3′ (reverse) for EP3*α*; 5′-CGG AAG TTC TGC CAG ATG ATG-3′ (forward) and 5′-CAG GGA AAC AGG TAC TGC AAT G-3′ (reverse) for EP3*β*; 5′-AGT TCT GCC AGG TAG CAA ACG-3′ (forward) and 5′-GCC TGC CCT TTC TGT CCA T-3′ (reverse) for EP3*γ*; 5′-CAT CCG TAA AGA CCT CTA TGC CAA C-3′ (forward) and 5′-ATG GAG CCA CCG ATC CAC A-3′ (reverse) for *β*-actin. The RT-PCR products were electrophoresed on 2.5% agarose gels and visualized with GelRed™ Nucleic Acid Gel Stain (Biotium, VWR, Leuven, Belgium).

### 2.8. Statistical Analysis

Data analyses were performed using GraphPad Prism 6.0 (GraphPad Software, San Diego, CA, USA). Data are expressed as mean ± S.E.M. or mean ± S.D. Statistical significance was assessed by one-way analysis of variance (ANOVA) followed by post hoc Tukey's multiple tests. Differences at *p* < 0.05 were considered to be statistically significant.

## 3. Results

### 3.1. PGE2- and EP2 Agonist-Induced Intracellular ROS Production in Differentiated NSC-34 Cells

**Figure 1:**
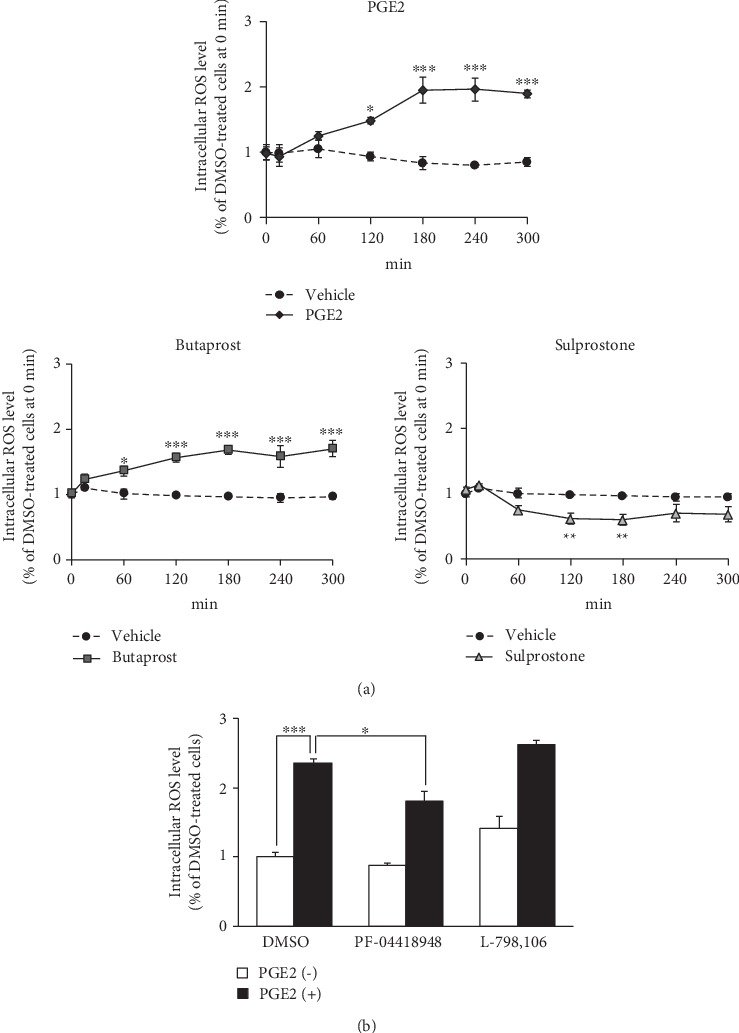
The intracellular ROS level in differentiated NSC-34 cells treated with PGE2 and EP agonist. (a) The intracellular levels of ROS were measured over time by monitoring DCF fluorescence intensity in differentiated NSC-34 cells incubated in vehicle (0.15% DMSO) or in the presence of PGE2 (80 *μ*M), butaprost (40 *μ*M), or sulprostone (40 *μ*M). Graphs show time courses of DCF fluorescence intensity in the four groups, respectively. Values represent means ± S.E.M. for four separate experiments. ^∗∗∗^*p* < 0.001, ^∗∗^*p* < 0.01, ^∗^*p* < 0.05 compared to vehicle-treated cells at each time point. (b) Differentiated NSC-34 cells were treated with 30 *μ*M PF-04418948 (an EP2 selective antagonist) or 10 *μ*M L798,106 (an EP3 selective antagonist) with or without 80 *μ*M PGE2 for 180 min. The graph illustrates the endpoint fluorescence values after these incubations. Each value represents the mean ± S.E.M. for four separate experiments. ^∗∗∗^*p* < 0.001, ^∗^*p* < 0.05 compared to vehicle-treated cells.

We first examined the effect of PGE2 on DCF fluorescence intensity as a marker of intracellular ROS production in differentiated NSC-34 cells preloaded with DCFH-DA. Intracellular ROS levels were markedly and time-dependently increased in cells treated with 80 *μ*M PGE2 (Figure 1(a)). The levels of intracellular ROS in these cells were significantly higher than those detected under vehicle treatment conditions after incubation for 120 to 300 min. We have reported previously that EP2 and EP3 are highly expressed in differentiated NSC-34 cells as well as motor neurons in the mouse spinal cord [21]. In order to clarify the type of EP receptor contributing to PGE2-induced ROS induction in differentiated NSC-34 cells, we investigated the effects of two well-characterized and selective EP agonists in the DCF fluorescence quantification assay. Exposure to an effective concentration range of butaprost (40 *μ*M), an EP2-selective agonist, resulted in a time-dependent increase of intracellular ROS production, and a statistically significant increase of DCF fluorescence was observed after incubation for 60 to 300 min (Figure 1(a)). In contrast, the level of intracellular ROS after treatment with 40 *μ*M sulprostone, an EP1/EP3 agonist, was transiently but significantly decreased after incubation for 120 to 180 min (Figure 1(a)). Moreover, DCF analysis showed that the generation of ROS caused by PGE2 at 80 *μ*M was attenuated significantly in the presence of PF-04418948, an EP2-selective antagonist, at 30 *μ*M (Figure 1(b)). In contrast, L-798,106, an EP3-selective antagonist, at 10 *μ*M did not suppress PGE2-induced ROS production (Figure 1(b)). PF-04418948 and L-798,106 did not change ROS formation in differentiated NSC-34 cells.

**Figure 2:**
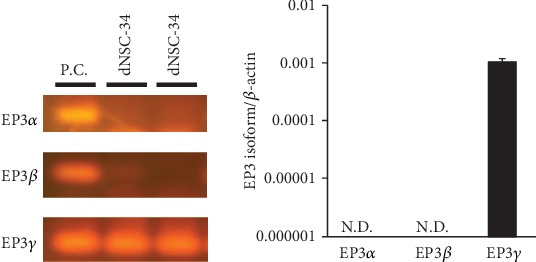
Characterization of mRNA expression for the EP3 isoform in motor neuron-like NSC-34 cells. Photographs show RT-PCR products of EP3*α* (101 bp), EP3*β* (63 bp), and EP3*γ* (61 bp). RT-PCR product amplified from differentiated NSC-34 cells (dNSC-34) and mouse hippocampus (positive control: P.C.) were electrophoresed on 2.5% agarose gels and visualized with GelRed™ Nucleic Acid Gel Stain. Representative data from at least four independent experiments are shown. Graphs show the expression profile of mRNAs for EP3 isoforms in differentiated NSC-34 cells. Expression of the mRNA for each EP3 isoform was normalized to the level of *β*-actin mRNA. Values represent the mean ± S.E.M. for four separate experiments. N.D.: not detected.

Mouse EP3 has three different isoforms (EP3*α*, EP3*β*, and EP3*γ*), and we have previously shown that expression of EP3*γ* mRNA is predominant in mouse motor neurons, whereas EP3*α* and EP3*β* are not detectable [26]. Therefore, we sought to identify the distribution of EP3 receptor isoforms in differentiated NSC-34 cells. As shown in Figure 2, semiquantitative real-time PCR demonstrated predominant expression of EP3*γ* in the cells, whereas EP3*α* and EP3*β* were undetectable.

### 3.2. N-Acetyl-L-Cysteine (NAC) Protects against PGE2- and EP2 Agonist-Induced Cell Death in Differentiated NSC-34 Cells

**Figure 3:**
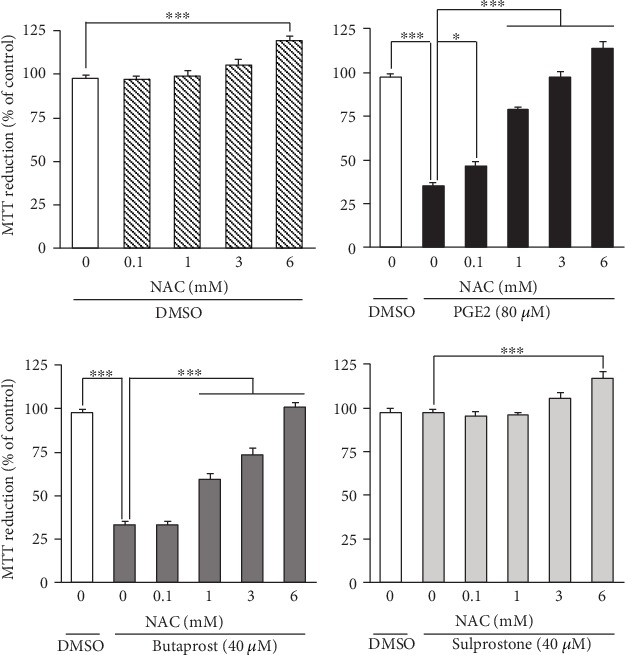
Effects of NAC pretreatment on PGE2- and EP agonist-induced cell death detected by MTT reduction assay. Differentiated NSC-34 cells were exposed to various concentrations of NAC for 4 h and then treated with vehicle (0.15% DMSO), 80 *μ*M PGE2, 40 *μ*M butaprost (an EP2 agonist), or 40 *μ*M sulprostone (an EP1/3 agonist) for 48 h. The viability of the cells was assessed by the MTT reduction assay. Results are expressed as percentages relative to the control (nontreated) cells. Values represent means ± S.E.M. for four separate experiments. ^∗∗∗^*p* < 0.001, ^∗∗^*p* < 0.01, ^∗^*p* < 0.05.

**Figure 4:**
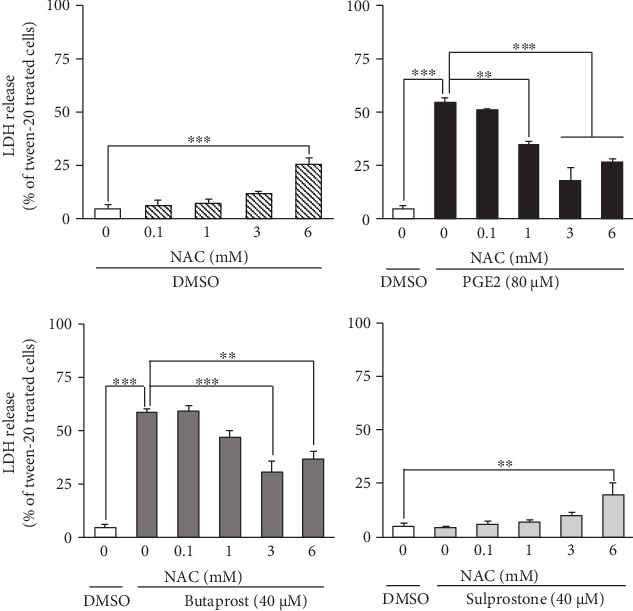
Effect of NAC pretreatment on PGE2- and EP agonist-induced LDH leakage from differentiated NSC-34 cells. Differentiated NSC-34 cells were exposed to various concentrations of NAC for 4 h, and then the cells were treated with vehicle (0.15% DMSO), 80 *μ*M PGE2, 40 *μ*M butaprost (an EP2 agonist), or 40 *μ*M sulprostone (an EP1/3 agonist) for 48 h. After exposure, the amount of LDH released into the medium was assayed as described in Experimental Procedures. Graphs show the relative levels of LDH in these cells. Values are calculated as percentages of LDH released relative to that of cells treated with 0.2% Tween-20. Values represent means ± S.E.M. for four separate experiments. ^∗∗∗^*p* < 0.001, ^∗∗^*p* < 0.01, ^∗^*p* < 0.05.

Next, we evaluated the effect of the antioxidant N-acetyl-L-cysteine (NAC) on PGE2- and butaprost-induced cell death using the MTT reduction assay (Figure 3) and LDH-based cytotoxicity assays (Figure 4). Consistent with previous results [21], exposure of the differentiated NSC-34 cells to 80 *μ*M PGE2 and 40 *μ*M butaprost for 48 h resulted in a significant decrease (35% and 33%, respectively) of cell survival in the MTT reduction assay (Figure 3). Unlike PGE2 and butaprost, exposure to 40 *μ*M sulprostone for 48 h had no significant effect within the concentration range for selective interaction with EP3. Pretreatment with NAC at 0.1–3 mM protected differentiated NSC-34 cells against PGE2-induced cell death in a concentration-dependent manner, although a high concentration of NAC (6 mM) yielded false-positive results in the MTT reduction assay due to their possible reducing activity on the MTT compound (Figure 3). Likewise, butaprost-induced cell death was rescued by pretreatment with NAC in a concentration-dependent manner, whereas NAC had no effect on the MTT levels in sulprostone-treated cells (Figure 3).

As shown in Figure 4, exposure to PGE2 and butaprost resulted in decreased cell viability (55% and 59%, respectively) when determined by LDH release assay, whereas sulprostone had no effect on the viability of these cells. Pretreatment with NAC at 0.1–3 mM protected these cells against PGE2- and butaprost-induced decreases in cell viability in a concentration-dependent manner (Figure 4), although the LDH assay also produced a false-positive result in cells treated with 6 mM NAC. In contrast, NAC had no effect on the level of LDH release in sulprostone-treated cells, as was the case in the MTT reduction assay (Figure 4).

**Figure 5:**
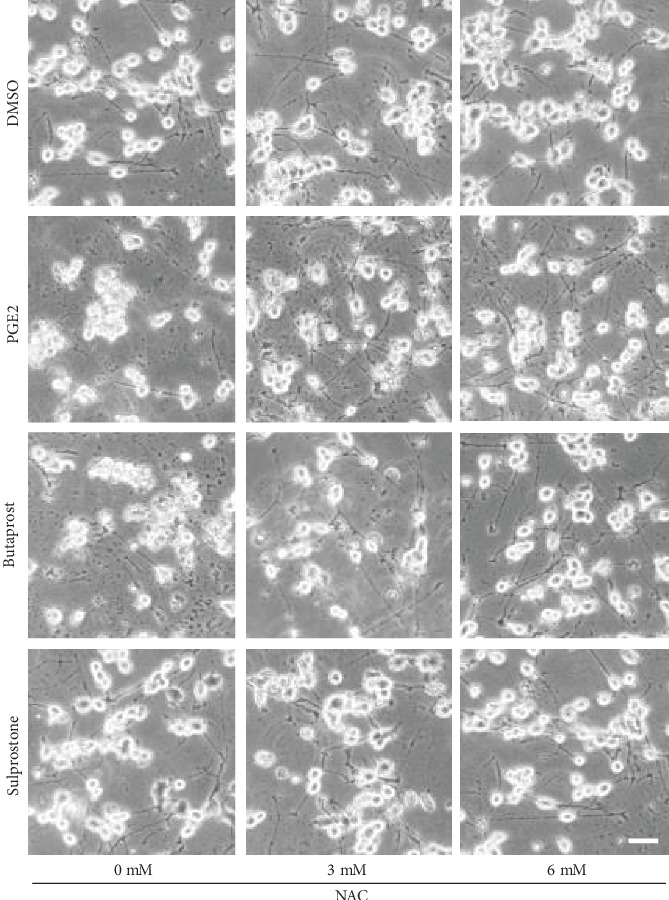
Phase-contrast photomicrographs of differentiated NSC-34 cells treated with PGE2 and EP agonists. Differentiated NSC-34 cells were treated with vehicle (0.15% DMSO), 80 *μ*M PGE2, 40 *μ*M butaprost (an EP2 agonist), or 40 *μ*M sulprostone (an EP1/3 agonist) in the presence or absence of NAC for 48 h. Cell morphology was observed using a phase-contrast microscope (IX71, Olympus, Tokyo, Japan). Photographs show typical phase-contrast microscopy images in each treatment group. Scale bar indicates 50 *μ*m.

Phase-contrast images showed no difference in morphology between the vehicle (DMSO)-treated cells and 6 mM NAC-treated cells within 48 h after the treatment (Figure 5). Exposure to PGE2 and butaprost, but not sulprostone, caused extensive alterations in cell morphology: the cells appeared clearly shrunken and rounded and were detached from the bottom of the culture plate (Figure 5). NAC pretreatment of these cells preserved their neuron-like cell morphology in a concentration-dependent manner upon treatment with PGE2 and butaprost (Figure 5).

**Figure 6:**
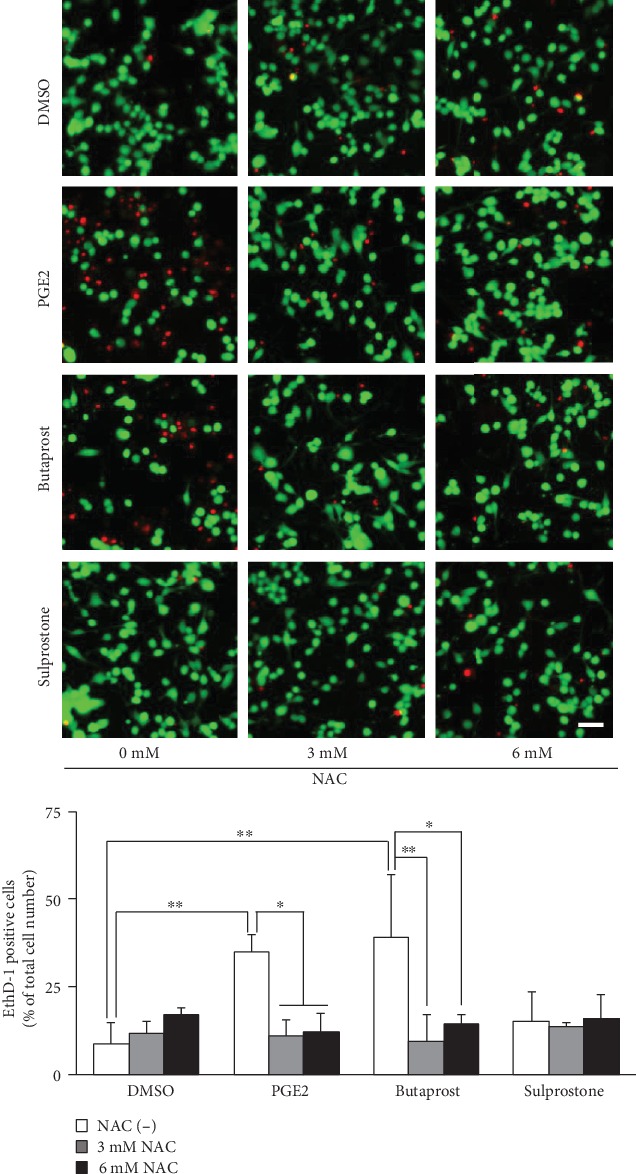
LIVE/DEAD staining of differentiated NSC-34 cells treated with PGE2 and EP agonists. Differentiated NSC-34 cells were treated with vehicle (0.15% DMSO), 80 *μ*M PGE2, 40 *μ*M butaprost (an EP2 agonist), or 40 *μ*M sulprostone (an EP1/3 agonist) in the presence or absence of NAC for 48 h. Photographs show typical fluorescence images of calcein-AM (green, live cells) and EthD-1 (red, dead cells) double staining in each treatment group. Scale bar indicates 50 *μ*m. Graphs show the percentage of EthD-1-positive dead cells in these cells. Each value represents the mean ± S.D. for three different experiments. ^∗∗^*p* < 0.01, ^∗^*p* < 0.05.

In order to investigate the change of cell mortality after treatment with NAC, Live/Dead assay was performed after various treatments. As shown in Figure 6, exposure of differentiated NSC-34 cells to 80 *μ*M PGE2 and 40 *μ*M butaprost, but not 40 *μ*M sulprostone, for 48 h led to an increase in the percentage of dead cells stained by EthD-1. Pretreatment with 3 mM and 6 mM NAC markedly reduced dead cells. Quantitative analysis showed that NAC significantly protected NSC-34 cells against PGE2- and butaprost-induced cell death.

### 3.3. NAC Suppresses the PGE2- and EP2 Agonist-Induced Increase in Cleaved Caspase-3 Protein in Differentiated NSC-34 Cells

**Figure 7:**
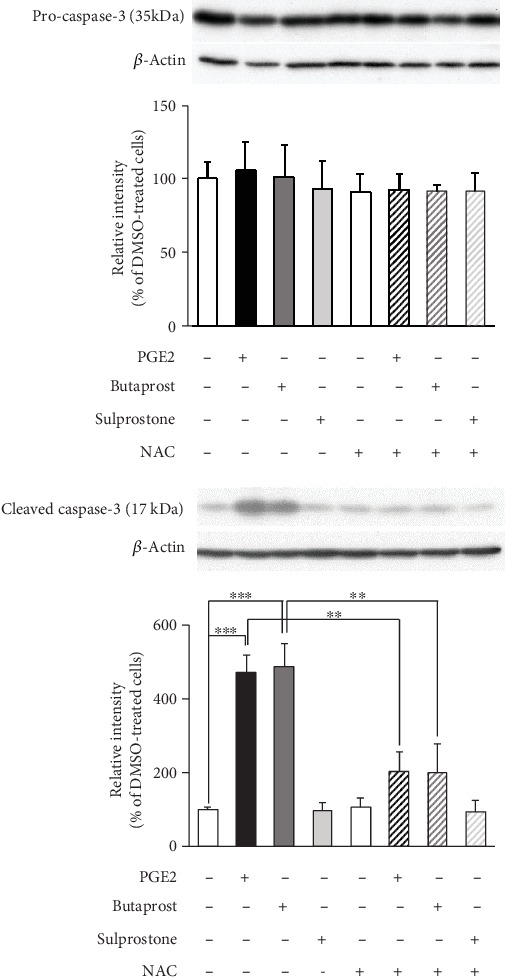
Effects of NAC pretreatment on PGE2- and EP agonist-induced caspase-3 cleavage in differentiated NSC-34 cells. Differentiated NSC-34 cells were treated with vehicle (0.15% DMSO), 80 *μ*M PGE2, 40 *μ*M butaprost (an EP2 agonist), or 40 *μ*M sulprostone (an EP1/3 agonist) in the presence or absence of 3 mM NAC for 8 h. Equal amounts of cell lysate (10 *μ*g) were analyzed by Western blotting using anti-pro-caspase-3 antibody, anti-cleaved caspase-3 antibody, and also with anti-*β*-actin antibody as an internal control. The level of pro-caspase-3 (35 kDa) and cleaved caspase-3 (17 kDa) was assessed by densitometric analysis, and quantitative results were expressed as the ratio of the band intensity of pro-caspase-3 or cleaved caspase-3 to the band intensity of *β*-actin. Each value represents the mean ± S.D. for four different experiments. N.D.: not detected. ^∗∗∗^*p* < 0.001, ^∗^*p* < 0.05.

PGE2 (5–25 *μ*M) has been reported to induce caspase-3 activation and apoptosis in cultured rat hippocampal neurons [27]. Therefore, we examined whether exogenously applied PGE2 and selective EP2 agonists are able to induce activation of caspase-3 in differentiated NSC-34 cells. Caspase-3 activation was detected by determining the level of caspase-3 cleavage fragments (17 kDa) using Western blotting. As shown in Figure 7, the levels of cleaved caspase-3 (17 kDa) were markedly increased after treatment of differentiated NSC-34 cells with PGE2 and butaprost, whereas sulprostone had no such effect. We also assessed the effects of NAC on the increased levels of cleaved caspase-3 after exposure to PGE2 and butaprost. Although NAC (3 mM) alone had no effect, the increase in the generation of cleaved caspase-3 induced by PGE2 and butaprost was significantly suppressed when the cells were pretreated with NAC (Figure 7). Unlike the results of cleaved caspase-3, the difference in the level of pro-caspase-3 was not detected following in any kind of treatment in differentiated NSC-34 cells shown in Figure 7.

### 3.4. Exogenously Applied Cyclic Adenosine Monophosphate (cAMP) Analog Induces Cell Death and Facilitates Intracellular ROS Production in Differentiated NSC-34 Cells

**Figure 8:**
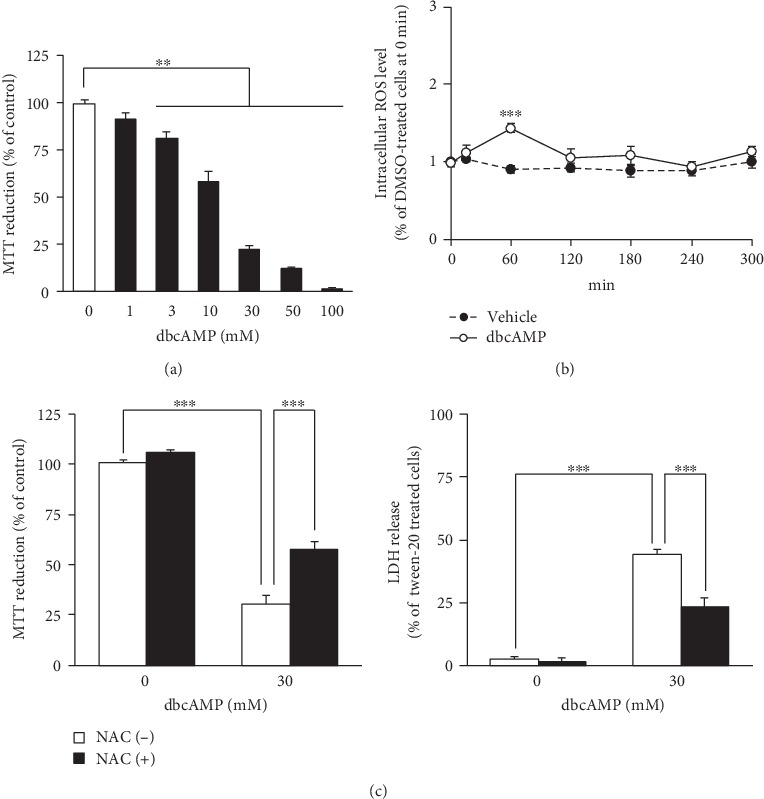
Exogenous application of cAMP analog induces cell death and facilitates intracellular ROS generation in differentiated NSC-34 cells. (a) Differentiated NSC-34 cells were incubated with various concentrations of dbcAMP or vehicle (distilled water) for 48 h. The viability of the cells was assessed by the MTT reduction assay. Graph shows effect of dbcAMP on MTT reduction activity in these cells. The results are expressed as a percentage relative to the control (nontreated) cells. Values represent means ± S.E.M. for four separate experiments. ^∗∗^*p* < 0.01. (b) The intracellular ROS levels were measured over time by monitoring DCF fluorescence intensity in differentiated NSC-34 cells incubated in vehicle (distilled water) or in the presence of dbcAMP (30 mM). Graph shows time courses of DCF fluorescence intensity in the treated cells. Values represent means ± S.E.M. for four separate experiments. ^∗∗∗^*p* < 0.001 compared to vehicle-treated cells at each time point. (c) Differentiated NSC-34 cells were exposed to vehicle (distilled water) or 3 mM NAC for 4 h, and then the cells were treated with vehicle (distilled water) or 30 mM dbcAMP for 48 h. The viability of the cells was assessed by the MTT reduction assay and LDH assay. Graphs show effect of dbcAMP on MTT reduction activity (left) and LDH release (right) in these cells. Results are expressed as a percentage relative to the control (nontreated) cells. Values represent means ± S.E.M. for four separate experiments. ^∗∗∗^*p* < 0.001.

It is generally accepted that EP2 is a Gs-coupled GPCR and increases the level of cyclic adenosine monophosphate (cAMP) [28]. In order to assess the functional significance of cAMP in the cell death induced by oxidative stress, we investigated the effect of dibutyryl-cAMP (dbcAMP), a cell-permeable cAMP analog, on cell survival and intracellular ROS production in differentiated NSC-34 cells. When differentiated NSC-34 cells were exposed to dbcAMP (1–100 mM) for 48 h, cell viability decreased in a concentration-dependent manner (Figure 8(a)). Statistically significant attenuation of cell viability was observed at doses of 3 mM and higher. Moreover, treatment with submaximal concentrations of dbcAMP (at 30 mM) transiently and significantly enhanced the production of intracellular ROS at 60 min (Figure 8(b)). Pretreatment with NAC at 3 mM abrogated the dbcAMP (30 mM)-induced decrease of MTT reduction activity and increase of LDH release (Figure 8(c)).

## 4. Discussion

PGE2 is known to be a potent proinflammatory mediator that is increased in postmortem brain tissue, cerebrospinal fluid, and serum from patients with sporadic ALS [6, 29] and in both the cerebral cortex and spinal cord in the G93A mutant SOD1 transgenic mouse model of ALS [7, 30]. A previous study from our laboratory showed that the PGE2-induced cytotoxicity is mediated by activation of EP2 in differentiated NSC-34 cells [21]. However, details of the mechanisms of cell injury triggered by PGE2 are not yet fully clear. The present results showed that PGE2 induced a dramatic increase of intracellular ROS generation in differentiated NSC-34 cells, strongly suggesting that PGE2-induced cell death in differentiated NSC-34 cells is attributable to intracellular ROS generation. We also showed that pretreatment of these cells with NAC reversed the decrease in MTT reduction activity, increases in the release of LDH, and the level of cleaved caspase-3 protein and cell death induced by PGE2 and butaprost. Although the level of cleaved caspase-3 increased in differentiated NSC-34 cells treated with PGE2, the decrease in pro-caspase-3 was not detected in these cells. Similar to our present results, several studies have reported increased level of cleaved caspase-3 without a decreased level in pro-caspase-3 [31, 32]. One possible explanation of this conflict is that a polyclonal antibody against pro-caspase-3 may have high affinity, so that no difference in the level of pro-caspase-3 may be detected. Present results also showed that the generation of cleaved caspase-3 was found in some extent in undifferentiated cells. The role of caspase-3 activity in promoting neuronal differentiation was demonstrated across a broad spectrum of cell lineages including olfactory sensory neurons, neural stem cell, and PC12 cells [33]. Unlike these cells, previous studies of our and other laboratories revealed that the generation of cleaved caspase-3 is observed in undifferentiated NSC-34 cells as well as in differentiated forms [22, 34]. Therefore, it seems that activation of caspase-3 is not responsible for the neuronal differentiation in NSC-34 cells. Similar to our present results, it has also been reported that the number of active caspase-3-positive cells is dramatically increased by several oxidative stress inducers such as hydrogen peroxide, tumor necrosis factor-*α*, and high doses of glutamate in differentiated NSC-34 cells [34]. On this basis, we conclude that PGE2 exerts apoptosis-inducing neurotoxicity via activation of the caspase-3 cascade, and that production of ROS acts upstream of the caspase-3 cascade to participate in the mechanism of cell death in motor neuron-like NSC-34 cells.

It has been shown that the pathological and physiological effects of PGE2 are mediated via four functionally related GPCRs, designated EP1–EP4 [28, 35]. Previous studies from our laboratory have demonstrated that EP2 and EP3 are highly expressed in differentiated NSC-34 cells as well as motor neurons in the mouse spinal cord, suggesting that differentiated NSC-34 cell is a suitable model for assessing the response to PGE2 in motor neurons [10, 21]. We have also shown that EP2 plays a key role in PGE2-induced cell death in differentiated NSC-34 cells [21]. Here, we further investigated the effects of well-characterized EP agonists and antagonists to clarify the mechanisms underlying PGE2-induced ROS production in differentiated NSC-34 cells. As in the case of PGE2, treatment of these cells with butaprost, a selective EP2 agonist, caused a time-dependent increase of ROS production. In contrast, application of an EP1/EP3 agonist, sulprostone, transiently decreased the levels of intracellular ROS. We also showed that an EP2-selective antagonist (PF-04418948) but not an EP3-selective antagonist (L-798,106) suppressed the PGE2-induced production of intracellular ROS. More importantly, generation of ROS was crucial to the actions of PGE2 and butaprost, as the antioxidant NAC (3 mM) suppressed the increase in the level of cleaved caspase-3 expression and the cell death induced by PGE2 and butaprost. These results suggest that PGE2-induced intracellular ROS production is mostly attributable to the activation of EP2, and not EP3, in differentiated NSC-34 cells.

Classically, EP2 has been shown to couple to Gs proteins and activate adenylate cyclase, leading to intracellular generation of cAMP and activation of cAMP-dependent protein kinases [28, 35]. It has been reported that the cAMP-dependent protein kinase A (PKA) signaling pathway is activated under hypoxic conditions and exacerbates hypoxia-induced ROS formation in PC-12 cells [36]. In contrast, activation of EP2 by butaprost has been reported to protect dopaminergic neurons against 6-hydroxydopamine (6-OHDA)-mediated oxidative stress in primary cultured neurons prepared from embryonic rat midbrain [37]. This neuroprotective effect of butaprost was also conferred by cAMP analogs and was blocked by PKA inhibitors, suggesting that the neuroprotection afforded by EP2 activation is mediated through cAMP-dependent PKA activity in these cells [37]. Recently, we demonstrated that PGE2 promotes the conversion of undifferentiated NSC-34 cells to motor neuronal cells by activating the EP2 subtype, and that an exogenously applied cAMP analog, dbcAMP, facilitates neurite outgrowth with no effect on cell proliferation in undifferentiated NSC-34 cells [22]. Unlike the situation in undifferentiated cells, the results of the present study showed that dbcAMP partially mimicked PGE2- and butaprost-induced intracellular ROS generation and cell death in differentiated NSC-34 cells. These results suggest that the cAMP signaling pathway is at least partly involved in PGE2-induced cytotoxicity in differentiated NSC-34 cells, and that CNS region, cell maturity, and differentiation could all be important in determining whether PGE2 causes salutary or detrimental effects in individual neurons. Although further study will be needed to identify the downstream mechanisms of EP2 activation operating in the cytotoxic effects of PGE2 on differentiated NSC-34 cells, the present study has newly revealed that activation of Gs-linked GPCRs evokes intracellular ROS generation in motor neuron-like cells.

The EP3 receptor has multiple isoforms generated through alternative mRNA splicing in the carboxyl tail of the EP3 receptor gene. Three mRNA splice variants of the EP3 receptor have so far been identified in the mouse: EP3*α*, EP3*β*, and EP3*γ* [28]. Previously, we have confirmed that EP3*γ* is the major expression isoform in the motor neurons of mice [26]. As is the case for the mRNA profile in mouse motor neurons, the present study showed that EP3*γ* is dominantly expressed in differentiated NSC-34 cells. Although the EP3*α* and EP3*β* isoforms couple exclusively to Gi protein, the EP3*γ* isoform couples to both Gs and Gi proteins [28]. Consistent with previous results from our laboratory [21], treatment of differentiated NSC-34 cells with sulprostone did not affect cell survival. We also found that intracellular ROS levels were slightly but significantly decreased in sulprostone-treated differentiated NSC-34 cells, and that PGE2-induced ROS production was not affected by the presence of L-798,106, an EP3-selective antagonist, in these cells. Thus, EP3 appeared not to play a role in PGE2-induced neurotoxicity in the cells.

Numerous studies have found evidence of increased oxidative stress in the pathogenesis of many neurodegenerative diseases, including ALS, Parkinson's disease, Alzheimer's disease, and Huntington disease [38]. We recently reported that the spinal cord PGE2 levels in the G93A mutant SOD1 transgenic mouse model of ALS at the early symptomatic stage tended to be increased relative to those in control mice [7]. Our present findings suggest that PGE2 may act as an inducer of oxidative stress in ALS-associated neuronal damage. In addition, we have previously revealed that the protein expression of mPGES-1, an inducible terminal synthase in PGE2 biosynthesis, is increased in the motor neurons of SOD1 mutant mice at the presymptomatic and early symptomatic stages, suggesting that PGE2 might be locally generated around motor neurons at 11 weeks of age [8]. Further studies are conducted to confirm the effects of PGE2 in the primary cultured motor neurons from mouse spinal cord and are needed to clarify the role of localized PGE2 in motor neuron oxidative stress in ALS model mice. Consistent with the important role of PGE2 suggested by our present findings, one recent study has shed new light on the relationship between PGE2 and oxidative stress using primary cultured mesencephalic neurons from mPGES-1-knockout mice [39]. Genetic deletion of mPGES-1 has been found to prevent dopaminergic neurodegeneration caused by 6-OHDA-induced oxidative stress and to inhibit 6-OHDA-induced PGE2 production both *in vitro* and *in vivo* [39]. Interestingly, exogenous application of PGE2 to mPGES-1-knockout neurons compensated for the deficiency of 6-OHDA-induced PGE2 production and abrogated 6-OHDA toxicity to almost the same extent as that seen in WT neurons [39]. These results suggest that mPGES-1 exacerbates 6-OHDA-induced dopaminergic neuronal death by enhancing oxidative stress via PGE2 production, thus increasing the vulnerability of neurons to oxidative stress through intracellular ROS generation in neurodegenerative diseases.

## 5. Conclusion

**Figure 9:**
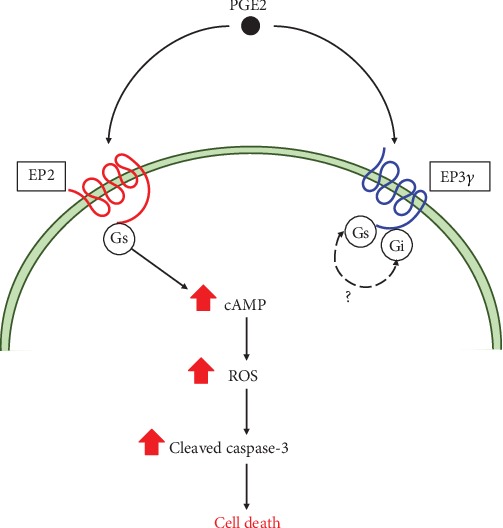
Proposed mechanism underlying PGE2-induced neurotoxicity in motor neuron-like NSC-34 cells. PGE2: prostaglandin E2; EP2: E-prostanoid receptor-2; EP3: E-prostanoid receptor-3; Gs: Gs protein; Gi: Gi protein; cAMP: cyclic adenosine monophosphate; ROS: reactive oxygen species.

In conclusion, the present study has demonstrated for the first time that PGE2 is an endogenous inducer of intracellular ROS, and that production of ROS induced by PGE2-EP2 receptor signaling is coupled to the caspase-3 cascade, the major pathway of apoptosis, in motor neuron-like NSC-34 cells, as shown in a summarized schema in Figure 9. These findings suggest that PGE2 is a lipid mediator with key links to inflammation and oxidative stress. Understanding this novel effect of PGE2 on oxidative stress in motor neuron-like cells may provide a potential target for the treatment of motor neuronal diseases such as ALS.

## Data Availability

The data used to support the findings of this study are available from the corresponding authors upon request.

## Abbreviations

aCSF: Artificial cerebrospinal fluid
cAMP: Cyclic adenosine monophosphate
ALS: Amyotrophic lateral sclerosis
dbcAMP: Dibutyryl-cAMP
DCF: Dichlorofluorescein
DCFH-DA: 2′,7′-Dichlorodihydrofluorescin diacetate
DMSO: Dimethyl sulfoxide
DP: D-prostanoid receptor
ECL: Enhanced chemiluminescence
EP: E-prostanoid receptor
EthD-1: Ethidium homodimer-1
FP: F-prostanoid receptor
FBS: Fetal bovine serum
GPCRs: G-protein-coupled receptors
IP: I-prostanoid receptor
LDH: Lactate dehydrogenase
mPGES-1: Microsomal PGE synthase-1
MTT: 3-(4,5-dimethylthiazol-2-yl)-2,5-diphenyl tetrazolium
NAC: N-acetyl-L-cysteine
6-OHDA: 6-Hydroxydopamine
PGD2: Prostaglandin D2
PGE2: Prostaglandin E2
PKA: cAMP-dependent protein kinase A
PVDF: Polyvinylidene difluoride
ROS: Reactive oxygen species
SDS: Sodium dodecyl sulfate
TP: Thromboxane-prostanoid receptors.

## References

[1] Liu J., Wang F. (2017). Role of neuroinflammation in amyotrophic lateral sclerosis: cellular mechanisms and therapeutic implications. *Frontiers in Immunology*.

[2] Rizzo F., Riboldi G., Salani S. (2014). Cellular therapy to target neuroinflammation in amyotrophic lateral sclerosis. *Cellular and Molecular Life Sciences*.

[3] Ferraiuolo L., Kirby J., Grierson A. J., Sendtner M., Shaw P. J. (2011). Molecular pathways of motor neuron injury in amyotrophic lateral sclerosis. *Nature Reviews. Neurology*.

[4] Peebles R. S. (2019). Prostaglandins in asthma and allergic diseases. *Pharmacology & Therapeutics*.

[5] Ricciotti E., FitzGerald G. A. (2011). Prostaglandins and inflammation. *Arteriosclerosis, Thrombosis, and Vascular Biology*.

[6] Almer G., Teismann P., Stevic Z., Halaschek-Wiener J., Deecke L., Kostic V. (2002). Increased levels of the pro-inflammatory prostaglandin PGE2 in CSF from ALS patients. *Neurology*.

[7] Miyagishi H., Kosuge Y., Takano A. (2017). Increased expression of 15-hydroxyprostaglandin dehydrogenase in spinal astrocytes during disease progression in a model of amyotrophic lateral sclerosis. *Cellular and Molecular Neurobiology*.

[8] Miyagishi H., Kosuge Y., Ishige K., Ito Y. (2012). Expression of microsomal prostaglandin E synthase-1 in the spinal cord in a transgenic mouse model of amyotrophic lateral sclerosis. *Journal of Pharmacological Sciences*.

[9] Shin J. H., Lee Y. A., Lee J. K. (2012). Concurrent blockade of free radical and microsomal prostaglandin E synthase-1-mediated PGE2 production improves safety and efficacy in a mouse model of amyotrophic lateral sclerosis. *Journal of Neurochemistry*.

[10] Kosuge Y., Miyagishi H., Yoneoka Y. (2018). Pathophysiological role of prostaglandin E2-induced up-regulation of the EP2 receptor in motor neuron-like NSC-34 cells and lumbar motor neurons in ALS model mice. *Neurochemistry International*.

[11] Parakh S., Spencer D. M., Halloran M. A., Soo K. Y., Atkin J. D. (2013). Redox regulation in amyotrophic lateral sclerosis. *Oxidative Medicine and Cellular Longevity*.

[12] Oskarsson B., Gendron T. F., Staff NP (2018). Amyotrophic lateral sclerosis: an update for 2018. *Mayo Clinic Proceedings*.

[13] Hald A., Lotharius J. (2005). Oxidative stress and inflammation in Parkinson’s disease: is there a causal link?. *Experimental Neurology*.

[14] Mhatre M., Floyd R. A., Hensley K. (2004). Oxidative stress and neuroinflammation in Alzheimer’s disease and amyotrophic lateral sclerosis: common links and potential therapeutic targets. *Journal of Alzheimer’s Disease*.

[15] Biswas S. K. (2016). Does the interdependence between oxidative stress and inflammation explain the antioxidant paradox?. *Oxidative Medicine and Cellular Longevity*.

[16] Hensley K., Mhatre M., Mou S. (2006). On the relation of oxidative stress to neuroinflammation: lessons learned from the G93A-SOD1 mouse model of amyotrophic lateral sclerosis. *Antioxidants & Redox Signaling*.

[17] Kondo M., Oya-Ito T., Kumagai T., Osawa T., Uchida K. (2001). Cyclopentenone prostaglandins as potential inducers of intracellular oxidative stress. *The Journal of Biological Chemistry*.

[18] Chen Y. C., Shen S. C., Tsai S. H. (2005). Prostaglandin D(2) and J(2) induce apoptosis in human leukemia cells via activation of the caspase 3 cascade and production of reactive oxygen species. *Biochimica et Biophysica Acta*.

[19] Rossi S. P., Windschuttl S., Matzkin M. E., Rey-Ares V., Terradas C., Ponzio R. (2016). Reactive oxygen species (ROS) production triggered by prostaglandin D2 (PGD2) regulates lactate dehydrogenase (LDH) expression/activity in TM4 Sertoli cells. *Molecular and Cellular Endocrinology*.

[20] Cashman N. R., Durham H. D., Blusztajn J. K. (1992). Neuroblastoma x spinal cord (NSC) hybrid cell lines resemble developing motor neurons. *Developmental Dynamics*.

[21] Miyagishi H., Kosuge Y., Yoneoka Y. (2013). Prostaglandin E2-induced cell death is mediated by activation of EP2 receptors in motor neuron-like NSC-34 cells. *Journal of Pharmacological Sciences*.

[22] Nango H., Kosuge Y., Miyagishi H., Sugawa K., Ito Y., Ishige K. (2017). Prostaglandin E2 facilitates neurite outgrowth in a motor neuron-like cell line, NSC-34. *Journal of Pharmacological Sciences*.

[23] Jang S., Yayeh T., Leem Y. H., Park E. M., Ito Y., Oh S. (2017). Concanavalin A induces cortical neuron apoptosis by causing ROS accumulation and tyrosine kinase activation. *Neurochemical Research*.

[24] Barber S. C., Higginbottom A., Mead R. J., Barber S., Shaw P. J. (2009). An in vitro screening cascade to identify neuroprotective antioxidants in ALS. *Free Radical Biology & Medicine*.

[25] Kosuge Y., Koen Y., Ishige K. (2003). S-allyl-L-cysteine selectively protects cultured rat hippocampal neurons from amyloid beta-protein- and tunicamycin-induced neuronal death. *Neuroscience*.

[26] Kosuge Y., Miyagishi H., Shinomiya T. (2015). Characterization of motor neuron prostaglandin E2 EP3 receptor isoform in a mouse model of amyotrophic lateral sclerosis. *Biological & Pharmaceutical Bulletin*.

[27] Takadera T., Shiraishi Y., Ohyashiki T. (2004). Prostaglandin E2 induced caspase-dependent apoptosis possibly through activation of EP2 receptors in cultured hippocampal neurons. *Neurochemistry International*.

[28] Sugimoto Y., Narumiya S. (2007). Prostaglandin E receptors. *The Journal of Biological Chemistry*.

[29] Ilzecka J. (2003). Prostaglandin E2 is increased in amyotrophic lateral sclerosis patients. *Acta Neurologica Scandinavica*.

[30] Klivenyi P., Kiaei M., Gardian G., Calingasan N. Y., Beal M. F. (2004). Additive neuroprotective effects of creatine and cyclooxygenase 2 inhibitors in a transgenic mouse model of amyotrophic lateral sclerosis. *Journal of Neurochemistry*.

[31] Stephan M., Edelmann B., Winoto-Morbach S. (2017). Role of caspases in CD95-induced biphasic activation of acid sphingomyelinase. *Oncotarget*.

[32] Zhou Q., Li Y., Jin J. (2012). Lx2-32c, a novel taxane derivative, exerts anti-resistance activity by initiating intrinsic apoptosis pathway in vitro and inhibits the growth of resistant tumor in vivo. *Biological & Pharmaceutical Bulletin*.

[33] Bell R. A. V., Megeney L. A. (2017). Evolution of caspase-mediated cell death and differentiation: twins separated at birth. *Cell Death and Differentiation*.

[34] Maier O., Böhm J., Dahm M., Brück S., Beyer C., Johann S. (2013). Differentiated NSC-34 motoneuron-like cells as experimental model for cholinergic neurodegeneration. *Neurochemistry International*.

[35] Narumiya S., Sugimoto Y., Ushikubi F. (1999). Prostanoid receptors: structures, properties, and functions. *Physiological Reviews*.

[36] Gozal E., Metz C. J., Dematteis M., Sachleben L. R., Schurr A., Rane M. J. (2017). PKA activity exacerbates hypoxia-induced ROS formation and hypoxic injury in PC-12 cells. *Toxicology Letters*.

[37] Carrasco E., Werner P., Casper D. (2008). Prostaglandin receptor EP2 protects dopaminergic neurons against 6-OHDA-mediated low oxidative stress. *Neuroscience Letters*.

[38] Li J., O W., Li W., Jiang Z. G., Ghanbari H. A. (2013). Oxidative stress and neurodegenerative disorders. *International Journal of Molecular Sciences*.

[39] Ikeda-Matsuo Y., Miyata H., Mizoguchi T. (2019). Microsomal prostaglandin E synthase-1 is a critical factor in dopaminergic neurodegeneration in Parkinson’s disease. *Neurobiology of Disease*.

